# Losartan does not inhibit cigarette smoke-induced lung inflammation in mice

**DOI:** 10.1038/s41598-019-51504-2

**Published:** 2019-10-21

**Authors:** M. L. Hepworth, S. L. Passey, H. J. Seow, R. Vlahos

**Affiliations:** School of Health and Biomedical Sciences, RMIT University, Bundoora, Victoria Australia

**Keywords:** Chronic obstructive pulmonary disease, Chronic inflammation

## Abstract

Chronic Obstructive Pulmonary Disease (COPD) is a progressive lung disease largely caused by cigarette smoking (CS) and is characterized by lung inflammation and airflow limitation that is not fully reversible. Approximately 50% of people with COPD die of a cardiovascular comorbidity and current pharmacological strategies provide little benefit. Therefore, drugs that target the lung and the cardiovascular system concurrently may be an advantageous therapeutic strategy. The aim of this study was to see whether losartan, an angiotensin-II AT1a receptor antagonist widely used to treat hypertension associated with cardiovascular disease, protects against CS-induced lung inflammation in mice. Male BALB/c mice were exposed to CS for 8 weeks and treated with either losartan (30 mg/kg) or vehicle daily. Mice were euthanized and bronchoalveolar lavage fluid (BALF) inflammation, and whole lung cytokine, chemokine and protease mRNA expression assessed. CS caused significant increases in BALF total cells, macrophages, neutrophils and whole lung IL-6, TNF-α, CXCL-1, IL-17A and MMP12 mRNA expression compared to sham-exposed mice. However, losartan only reduced CS-induced increases in IL-6 mRNA expression. Angiotensin-II receptor expression was reduced in lung tissue from CS-exposed mice. In conclusion, losartan did not inhibit CS-induced BALF cellularity despite reducing whole lung IL-6 mRNA and Ang-II receptor expression.

## Introduction

Chronic Obstructive Pulmonary Disease (COPD) is a devastating lung disorder most commonly caused by repeated inhalation of cigarette smoke (CS)^1^. The diseases encompasses emphysema, chronic obstructive bronchiolitis, and mucus-plugging of the airways^1,2^. There is also increased lung inflammation consisting of macrophages, neutrophils, and lymphocytes^3^. It is recognised that macrophages and neutrophils are major drivers of the pathology observed in COPD. Cigarette smoke induces the release of inflammatory factors such as tumour necrosis factor (TNF)-α, interleukin (IL)-6, C-C Motif Chemokine Ligand (CCL) 2, and reactive oxygen species (ROS) from alveolar macrophages^2,3^. Similarly, the number of neutrophils in the bronchoalveolar lavage fluid (BALF) and lung tissue of COPD patients in also increased^2,3^. These neutrophils secrete multiple proteases in the lungs of patients which then destroy the lung tissue leading to emphysema^3,4^.

In addition to lung inflammation and pathology, patients often develop other chronic medical conditions (called comorbidities) including cardiovascular disease which further impact the patient’s health and survival^5–7^. It is well recognised that the majority of the burden and healthcare cost in COPD is due to treating the various comorbidities which affect disease morbidity and mortality^8^. There is evidence that up to 50% of all COPD deaths are because of a cardiovascular comorbidity. In addition, prospective clinical trials have not identified a pharmaceutic that can decrease the risk of death in COPD^7,9–12^. There are studies showing that cardiovascular disease accounts for a significant proportion of first and second hospitalizations (42% and 44%, respectively) in COPD^13^ and that arrhythmias were associated with significant in-hospital mortality of COPD patients^14,15^.

Current COPD therapy is relatively ineffective^16,17^ and since a significant number of COPD patients will die of a cardiovascular comorbidity, drugs that target the lung and the cardiovascular system concurrently may be an advantageous therapeutic strategy. Epidemiological studies have suggested that angiotensin converting enzyme (ACE) inhibitors which are used to treat hypertension and cardiac failure (common comorbidities of COPD), may benefit COPD, by reducing mortality and exacerbations^18–21^. In addition, COPD patients on angiotensin II receptor blockers (ARBs) had reduced rates of pneumonia and mortality compared to COPD patients on ACE inhibitors^22^. The beneficial actions of ACE inhibitors in COPD may go beyond them attenuating pulmonary hypertension because angiotensin II (ang II) has pro-inflammatory effects^23^. In fact, ARBs reduce hyperinflation in subjects with COPD^24^ and attenuate CS-induced lung injury in mice^25^. Moreover, ACE inhibitors and ARBs have been shown to slow the progression of percent emphysema, especially among former smokers^26^. Randomized clinical trials of ARBs are underway to evaluate their effect on the progression of emphysema in COPD (https://clinicaltrials.gov/ct2/show/NCT02696564).

In this study, we examined whether losartan, an angiotensin-II AT_1_ receptor antagonist widely used to treat hypertension associated with cardiovascular disease, would significantly reduce lung inflammation in mice chronically exposed to CS. Identification of drugs that target the lung and the cardiovascular system concurrently may be advantageous for the treatment of COPD and comorbid cardiovascular disease.

## Methodology

### Mice

Specific pathogen-free male BALB/c mice (7 weeks of age) were obtained from the Animal Resource Centre Pty. Ltd. (Perth, Australia). Mice were housed in clean micro-isolator cages at 21 °C on a 12-hour day/night cycle with access to as much food or water as desired. After an acclimatisation period of four days, mice were weighed and organised into groups to assure the same average starting weight in each group. CS-exposed mice had access to as much food or water as desired. Mice were individually weighed 5 days per week (Monday to Friday). All experiments were approved by the Animal Ethics Committee of RMIT University (Ethics ID1521) and performed in accordance with the guidelines of animal experimentation established by the National Health and Medical Research Council of Australia.

### Losartan treatment

Mice were treated with either losartan (Losartan Potassium, Sigma-Aldrich Co., Australia) or vehicle (water). Losartan Potassium was dissolved in the drinking water and mice had *ad libitum* access (0.25 g/L to correspond to a dose of 30 mg/kg). Losartan was prepared in fresh water daily for the duration of the protocol.

### Cigarette smoke exposure

Mice were placed in an 18-L perspex chamber (The Plastic Man, Huntingdale, Victoria, Australia) in a class II biosafety cabinet (AES Environmental Pty Ltd, Melbourne, Victoria, Australia) and exposed to CS generated from three cigarettes (Winfield Red, ≤16 mg tar, ≤15 mg carbon monoxide, ≤1.2 mg nicotine; Philip Morris, Moorabbin, Australia) spaced evenly over 1 hour, and carried out three times per day (08:00, 11:00, and 14:00 h) for five days a week for 8 weeks as previously published^27^. Cigarette smoke was generated in 50-ml tidal volumes over 10 s as previously published^27^. The mean total suspended particulate mass concentration in the chamber containing CS was ~420 mg m^−3^; this level of CS exposure causes increases in blood carboxyhemoglobin comparable to that observed in human smokers^27,28^. Sham-exposed mice were handled the same way but were exposed to room air. At the end of the protocol, mice were euthanised with an overdose of anaesthetic (Lethabarb; 60 mg/kg; Virbac Pty. Ltd., Australia) via intraperitoneal injection (ip), and the analyses described below performed.

### Bronchoalveolar lavage and tissue collection

Lungs were lavaged *in situ* with a 400 µl aliquot of PBS, followed by three 300 µl aliquots as previously described^27,29^. Approximately 1 ml of bronchoalvealor avage fluid (BALF) was recovered per mouse. The total number of viable cells in the BALF was determined, cytospins were prepared using 50 μl of BALF (~5 × 10^4^ cells). Cytospin cells (500 per slide) were fixed in Kwik-Diff Reagent 1 (Thermo-Fisher, Massachusetts, USA), and then stained with Hemacolor® Rapid Solution 2 and 3 (Merck Millipore, Massachusetts, USA), and discerned into macrophages, neutrophils and lymphocytes by standard morphological criteria. The remaining BALF was centrifuged at 660 g for 5 min at 4 °C to collect the supernatant and stored at −80 °C until required. 5 mL of chilled PBS was used to perform a right ventricular perfusion of the heart to clear the lungs of blood. Lungs were then quickly excised, weighed, snap-frozen in liquid nitrogen, and stored at −80 °C until required. The liver, kidneys, retroperitoneal fat, and testicular fat were rapidly dissected, weighed, snap-frozen in liquid nitrogen and stored at −80 °C until required.

### RNA extraction, cDNA synthesis and quantitative real time PCR

The whole lung from each mouse was crushed into a powder using a mortar and pestle with liquid nitrogen to avoid thawing. Two small scoops of tissue powder (~15 mg) were then transferred into a 1.7 mL Eppendorf (Germany) tube containing 600 μL RLT Lysis buffer (RNeasy® Mini Kit 250, Qiagen, Germany) supplemented with a 1:100 dilution of β-Mercaptoethanol (1000 × 2-Mercaptoethanol, Life Technologies Co., USA). The tissue was homogenised by passing it through a 21 g needle (Terumo Co., Japan) and 1 mL syringe (Livingstone International Pty. Ltd., Australia) 5–10 times. The Eppendorf tube was then centrifuged at 16,000 g for 3 minutes on a Sigma Laborzentrifugen 1–14 centrifuge (John Morris Scientific Pty. Ltd., Australia) and supernatant was collected. The RNA was then purified with an RNeasy Mini Kit 250 (Qiagen, Germany) and the concentration and purity of the extracted RNA determined with a NanoDrop 2000 spectrophotometer (Thermo Fisher Scientific Inc., USA). Single strand cDNA was synthesised by reverse transcription using the High Capacity RNA-to-cDNA Kit (Applied Biosystems, USA) and 1 μg of RNA used for cDNA synthesis.

Quantitative real time polymerase chain reaction (RT-qPCR) was performed using mouse-specific TaqMan Gene Expression Assays (Table 1, Applied Biosystems, Australia), with Taqman Fast Advanced Master Mix (Thermo Fisher Scientific Inc., Australia) on a QuantStudio 7 Flex Real-Time PCR System (Applied Biosystems, Australia). All samples were assayed in triplicate, and the assay was run for 40 cycles. All samples were standardised to GAPDH (housekeeping gene) and fold-change was determined in comparison to the sham vehicle group. Results were analysed via the 2−ΔΔCT method^27,29–31^.

**Table 1:** List of Taqman Gene Expression Assays.

Gene (predesigned assays)	Accession number	Assay ID
GAPDH	NM_008084.2	Mm99999915_g1
IL-6	NM_031168.1	Mm00446190_m1
TNF-α	NM_013693.3	Mm00443258_m1
CXCL1	NM_008176.3	Mm04207460_m1
CCL2	NM_011333.3	Mm00441242_m1
IL-17A	NM_010552.3	Mm00439618_m1
Agtr1a	NM_177322.3	Mm01166161_m1
Agtr2	NM_007429.5	Mm01341373_m1
Agt	NM_007428.3	Mm00599662_m1
Mmp12	NM_008605.3	Mm00500554_m1

All Taqman Gene Expression are pre-designed assays developed by Applied Biosystems. The accession number, and assay ID number for each number are listed.

### Statistical analyses

Data are expressed as mean ± standard error of the mean (SEM) and *n* denotes the number of mice used in each group. Statistical differences were decided by two-way ANOVA followed by Sidak’s post-hoc test for multiple comparisons. GraphPad Prism for Windows (Version 8.0.1, GraphPad Sofware, Inc., USA) was used for all analyses and *P* < 0.05 was used to indicate statistical significance.

## Results

### Effect of losartan on CS-induced BALF inflammation and lung weight

**Figure 1:**
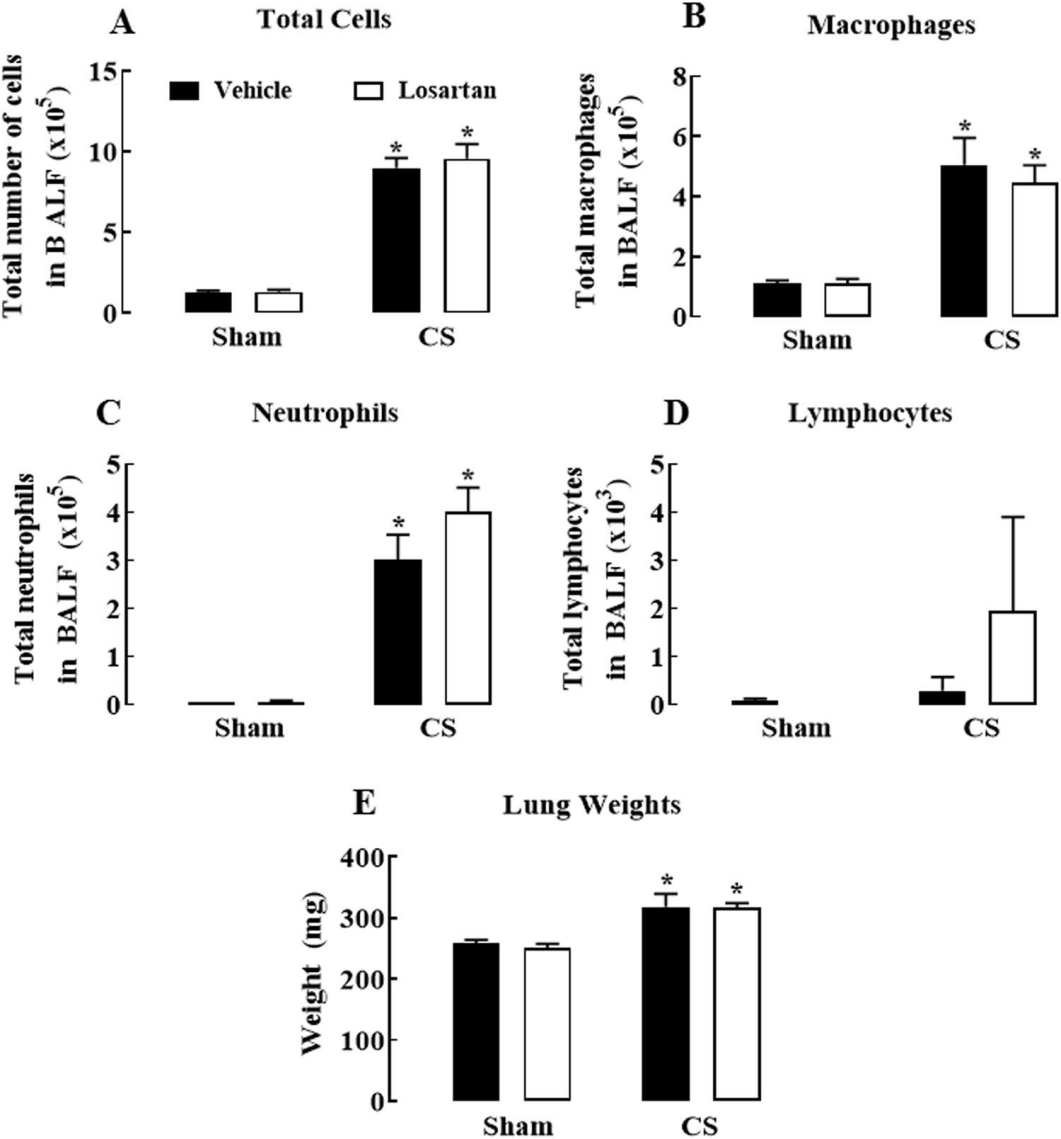
Effect of cigarette smoke exposure and losartan treatment on inflammatory cell numbers in bronchoalveolar lavage fluid (BALF) and lung weight. Number of total cells (**A**), macrophages (**B**), neutrophils (**C**) and lymphocytes (**D**) in BALF, and lung weight (**E**). Mice were exposed to cigarette smoke (CS) or room air (sham) for 8 weeks and treated with losartan (30 mg/kg) or vehicle (saline) administered in the drinking water. Data are expressed as mean ± SEM (n = 9–10 mice per group) and analysed by two-way ANOVA with Sidak’s post hoc test for multiple comparisons. *P < 0.05 compared to sham (vehicle).

Cigarette smoke exposure significantly increased the number of BALF total cells, macrophages and neutrophils (*P* < 0.05) (Fig. 1A–C) but did not affect lymphocyte numbers (*P* > 0.05) (Fig. 1D). However, losartan had no effect on CS-induced BALF cellularity (*P* > 0.05). In addition, CS significantly increased the lung weight compared to sham-exposed vehicle mice (*P* < 0.05) and losartan did not affect this response (*P* > 0.05) (Fig. 1E).

### Effect of losartan on lung tissue gene expression

To further characterise the inflammatory response to CS and losartan treatment, the mRNA expression of proinflammatory cytokines (IL-6, TNF-α), chemokines (C-X-C Motif Chemokine Ligand [CXCL]-1, C-C Motif Chemokine Ligand 2 [CCL2], IL-17A) and proteases (Matrix Metalloproteinase 12 [MMP12]) was measured in whole lung tissue.

**Figure 2:**
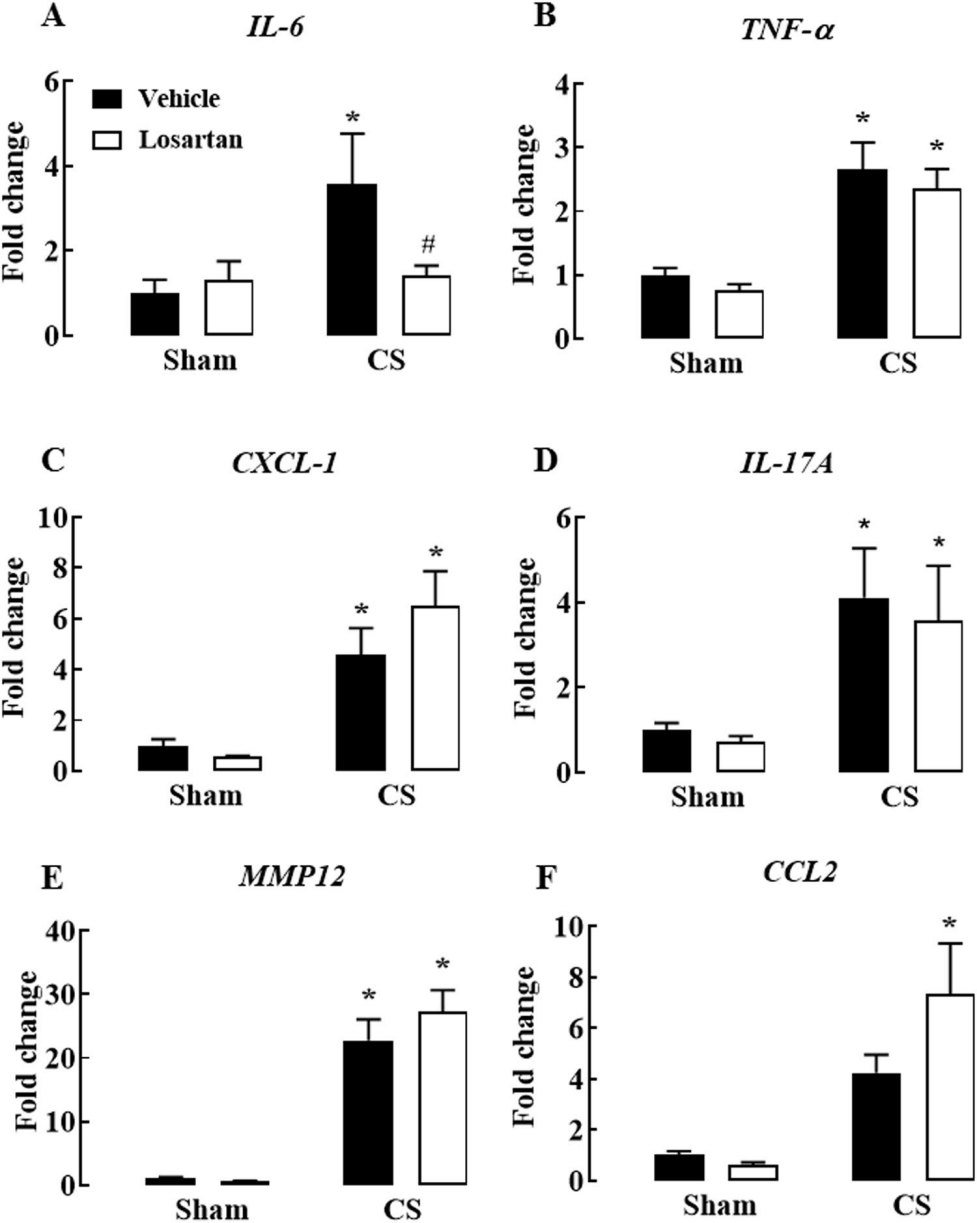
Effect of cigarette smoke exposure and losartan treatment on inflammatory cytokine and chemokine mRNA expression in whole lung tissue. Gene expression of interleukin-6 (**A**), tumor necrosis factor-α (**B**), chemokine ligand 1 (**C**), interleukin-17A (**D**), matrix metalloproteinase 12 (**E**) and chemokine ligand 2 (**F**) in lung tissue. Fold change was determined in comparison to sham (vehicle), after standardising samples to GAPDH (housekeeping gene). Mice were exposed to cigarette smoke (CS) or room air (sham) for 8 weeks and treated with losartan (30 mg/kg) or vehicle (saline) administered in the drinking water. Data are expressed as mean ± SEM (n = 4–10 mice per group) and analysed by two-way ANOVA with Sidak’s post hoc test for multiple comparisons. *P < 0.05 compared to sham (vehicle), ^#^*P* < 0.05 compared to smoke (vehicle).

Cigarette smoke exposure caused a significant increase in the mRNA expression of the proinflammatory cytokines, IL-6 and TNF-α, in the lung of CS-exposed mice when compared to sham vehicle mice (*P* < 0.05) (Fig. 2A,B). Losartan caused a significant decrease in CS-induced increases in IL-6 mRNA expression (*P* < 0.05) but was without effect on the increased TNF-α mRNA expression (*P* > 0.05) (Fig. 2A,B).

Cigarette smoke exposure caused a significant increase in CXCL-1, IL-17A and MMP12 mRNA expression compared to sham + vehicle mice (*P* < 0.05) (Fig. 2C–E) but losartan did not affect these increases in mRNA expression (*P* > 0.05) (Fig. 2C–E). Cigarette smoke did not cause a statistically significant increase in CCL2 mRNA expression although it was a 5-fold increase compared to sham-exposed vehicle mice (*P* > 0.05) (Fig. 2F). A significant increase in CCL2 expression was observed in CS-exposed losartan-treated mice compared to the sham + vehicle mice (*P* < 0.05) (Fig. 2F).

**Figure 3:**
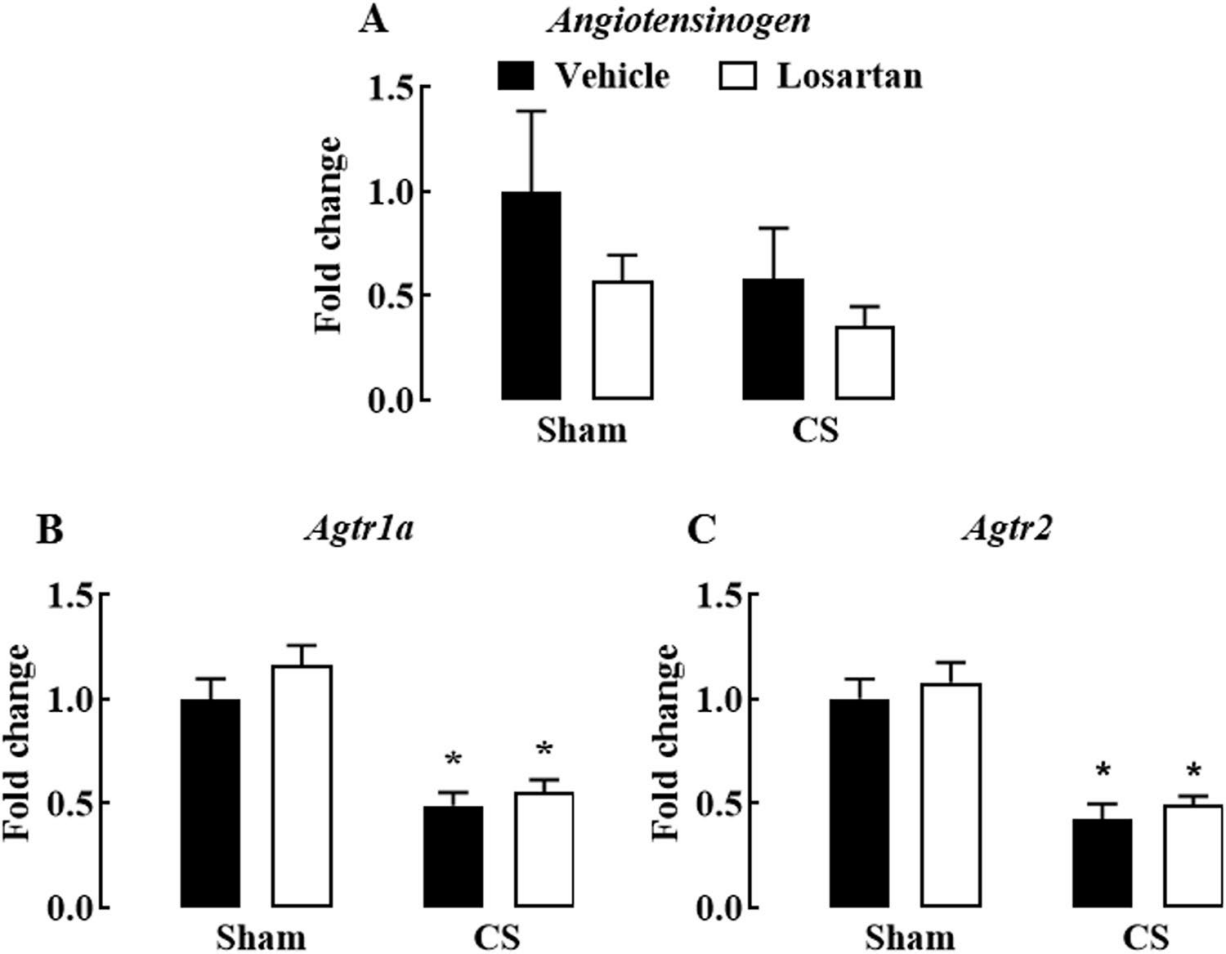
Effect of cigarette smoke exposure and losartan treatment on mRNA expression of components of the Renin-Angiotensin System in whole lung tissue. Gene expression of angiotensinogen (**A**), angiotensin-II AT1a (Agtr1a) receptor (**B**), and angiotensin-II AT2 (Agtr2) receptor. (**C**) Relative expression was determined in comparison to sham (vehicle), after standardising samples to GAPDH (housekeeping gene). Mice were exposed to cigarette smoke (CS) or room air (sham) for 8 weeks and treated with losartan (30 mg/kg) or vehicle (saline) administered in the drinking water. Data are expressed as mean ± SEM (n = 9–10 mice per group) and analysed by two-way ANOVA with Sidak’s post hoc test for multiple comparisons. *P < 0.05 compared to sham (vehicle).

The mRNA expression of components of the renin angiotensin system (RAS), including angiotensinogen and the two angiotensin-II receptors, Agtr1a and Atr2, were also measured to determine the RAS-specific effects of losartan. Cigarette smoke exposure and losartan had no effect on mRNA expression of angiotensinogen (*P* > 0.05) (Fig. 3A). However, CS exposure caused a significant reduction in the expression of both Agtr1a and Agtr2 compared to sham + vehicle mice (*P* < 0.05) (Fig. 3B,C); however, losartan was without effect on these responses (*P* > 0.05) (Fig. 3B,C).

### Effect of cigarette smoke exposure and losartan treatment on body, kidney, liver and white adipose tissue weights

**Figure 4:**
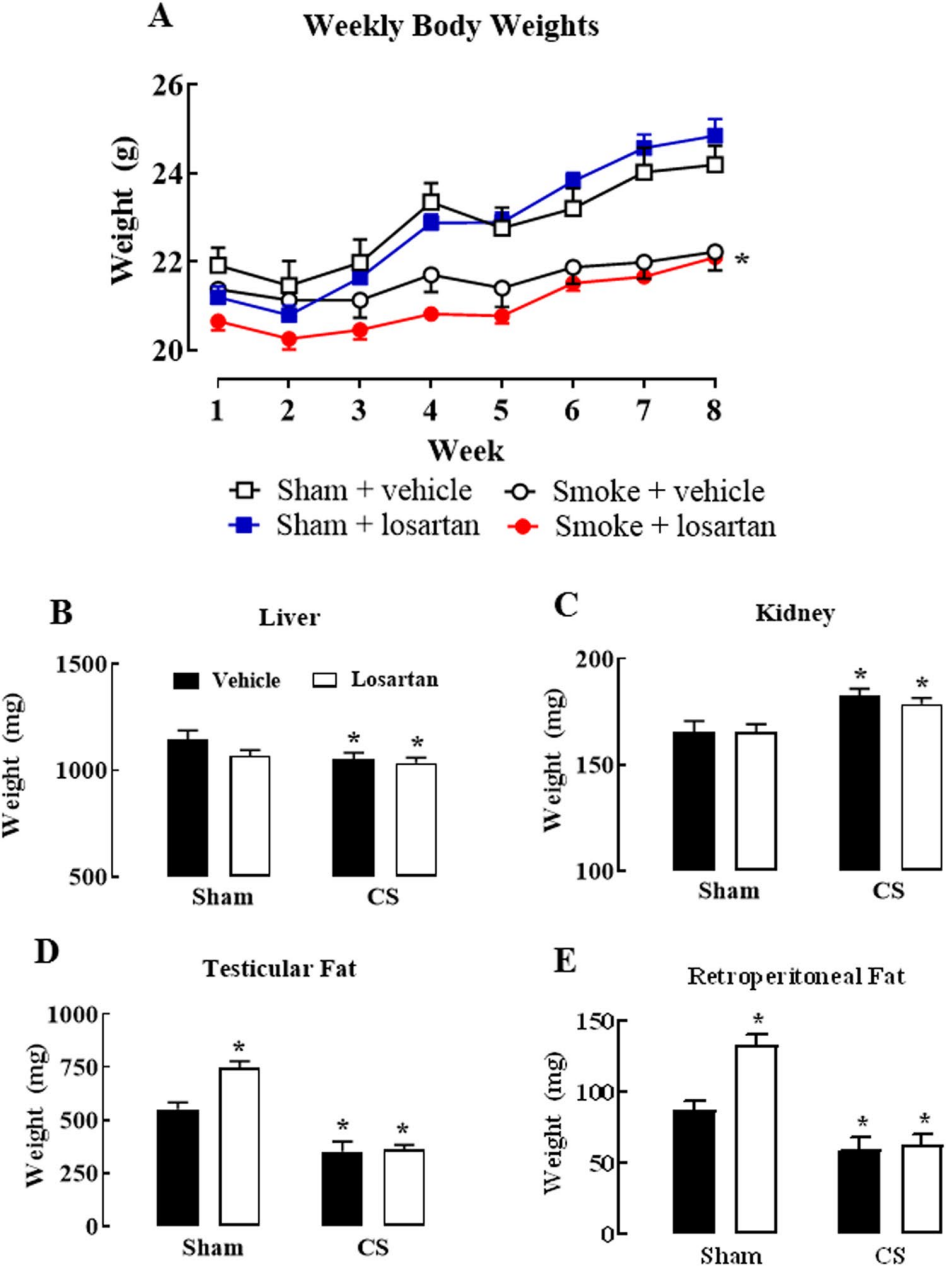
Effect of cigarette smoke exposure and losartan treatment on body, organ and white adipose tissue weights. Progressive body weight of mice across the experimental period (**A**) and liver (**B**), kidney (**C**), testicular fat (**D**) and retroperitoneal fat (**E**) weights at the end of the 8-week cigarette smoke exposure protocol. Mice were exposed to cigarette smoke (CS) or room air (sham) for 8 weeks and treated with losartan (30 mg/kg) or vehicle (saline) administered in the drinking water. Data are expressed as mean ± SEM (n = 9–10 mice per group) and analysed by two-way ANOVA with Sidak’s post hoc test for multiple comparisons. *P < 0.05 compared to sham (vehicle).

To understand the effect of both CS and losartan on body weight, mice were monitored for the duration of the protocol. The body weight of CS-exposed mice significantly reduced compared to sham-exposed vehicle mice (*P* < 0.05), but losartan did not affect this response (*P* > 0.05) (Fig. 4A).

Various organs (liver, kidney) and white adipose tissue (testicular and retroperitoneal fat) were weighed to further explore the effect of CS exposure and losartan treatment on body weight. Liver mass was significantly decreased in the CS-exposed losartan-treated group when compared to the sham vehicle group (*P* < 0.05) (Fig. 4B). The kidneys of CS-exposed mice were significantly heavier than those in the sham-exposed vehicle group (*P* < 0.05) (Fig. 4C). However, losartan did not affect this change in either the sham or CS groups (*P* > 0.05) (Fig. 4C). CS-exposed mice had significantly reduced testicular and retroperitoneal fat (*P* < 0.05), independent of losartan treatment (*P* > 0.05) (Fig. 4D,E). However, losartan-treated sham mice had significantly increased testicular and retroperitoneal fat compared to sham vehicle mice (*P* > 0.05) (Fig. 4D,E).

### Average food and water consumption

**Figure 5:**
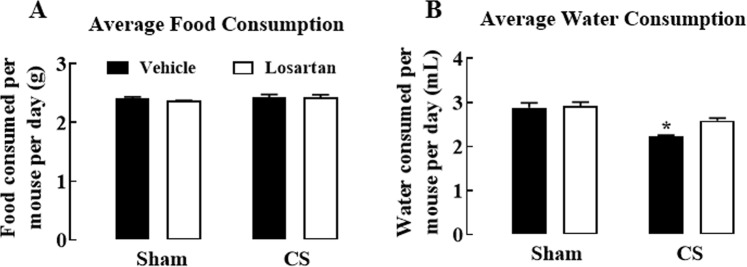
Average food and water consumption during cigarette smoke exposure and losartan treatment. Average food consumed (**A**), and average water consumed (**B**) per mouse per day. Mice were exposed to cigarette smoke (CS) or room air (sham) for 8 weeks and treated with losartan (30 mg/kg) or vehicle (saline) administered in the drinking water. Sham groups were pair fed, receiving the same amount of food that the relevant smoke group ate in the previous 24 hours. Food intake of the CS-exposed groups was measured daily at approximately the same time to calculate the amount of food the sham groups would receive. Water intake was measured daily by calculating the difference in weight of water bottles in all cages across 24 hours. Data expressed as mean ± SEM (n = 9–10 mice per group) and analysed by two-way ANOVA with Sidak’s post hoc test for multiple comparisons. ^*^P < 0.05 compared to sham (vehicle).

To investigate the effect of CS exposure and losartan on appetite, food and water intake was measured in the CS groups daily throughout the 8 weeks. There were no changes in the food consumed in any treatment group *(P* > 0.05) (Fig. 5A). Water consumption was measured daily to determine the effects of CS and losartan treatment on water intake. Vehicle-treated mice exposed to CS drank significantly less water when compared to sham-exposed vehicle mice (*P* < 0.05) (Fig. 5B).

## Discussion

COPD consists of not only a persistent lung inflammation that leads to reduced airflow but also systemic inflammation that leads to comorbidities including cardiovascular disease. Studies have implicated the renin angiotensin system (RAS) in the development of pulmonary and extrapulmonary disorders in COPD thus highlighting the potential therapeutic benefit of RAS blockade in subjects with COPD^32^. Therefore, we examined whether the angiotensin-II AT1 receptor antagonist losartan would significantly reduce lung inflammation in mice chronically exposed to CS and thus be a promising medication for the treatment of COPD and comorbid cardiovascular disease. We found that losartan did not inhibit CS-induced BALF cellularity despite reducing whole lung IL-6 mRNA and angiotensin II receptor mRNA expression.

Consistent with our previous studies^33,34^, we found in this study that mice exposed to CS had a significant increase in total inflammatory cells in the BALF, which was largely attributed to increased macrophages and neutrophils. Human studies have also shown that COPD patients have increased macrophages in BALF, sputum, airways and lung parenchyma compared to healthy individuals^2^. To determine the cause of this inflammation, the mRNA expression of chemokines in the lung tissue were explored. CXCL1, involved in neutrophil recruitment^35^, was markedly elevated in CS-exposed mice. IL-17A, a neutrophil chemotactic factor, has been shown to be elevated in the small airway epithelium in COPD subjects compared to non-COPD smokers and non-smokers^36^. In this study, IL-17A mRNA expression was markedly elevated in lung tissue from CS-exposed mice, which is in accord with our previous work demonstrating a pathogenic role of IL-17A in COPD^37,38^. Wang *et al*. have shown that CCL2 levels are elevated in AECOPD patients when compared to healthy individuals^39^. We observed a 5-fold increase in whole lung CCL2 expression in CS-exposed mice, though this was not statistically significant. IL-6 and TNF-α are two pro-inflammatory cytokines that are elevated in lungs of subjects with COPD^40^. In our chronic CS-exposure model, these cytokines had significantly increased mRNA expression in CS-exposed mice compared to sham vehicle mice. Moreover, the whole lung weighed significantly more in the CS-exposed mice when compared to sham vehicle mice, most likely due to the increased inflammatory response causing oedema.

It is well known that proteases break down lung tissue to cause emphysema and hence play an important role in COPD^41^. Studies have shown increased concentrations of MMP-1 and MMP-9 in BALF of subjects with emphysema^42^. In addition, increases in MMP-9 activity in lung parenchyma has also been demonstrated in people with emphysema^43^. Normal smokers have more MMP-9 in their alveolar macrophages than normal subjects^44^, and those with COPD have even greater levels^45^. A pathogenic role of MMP-12 has also been demonstrated in COPD whereby emphysema caused by CS was abolished in MMP-12^−/−^ mice^46^. Proteolytic enzymes such as MMP-2, MMP-9, MMP-12 are also released from alveolar macrophages as are cathepsin K, L, and S, which all cause destruction of the lung parenchyma^2,41^. The elevated levels of MMP-12 mRNA in our study was likely due to the accumulation of macrophages.

In addition to lung inflammation, mRNA expression of the various RAS components were investigated in the lung to determine the impact of CS. The two angiotensin-II receptors (Agtr1a and Agtr2) have been shown to be present on epithelial cells in the lung, with localisation of Agtr1a mainly on the parenchyma^47^. Podowski *et al*.^25^ demonstrated no statistically significant change in Agtr1a expression in lung tissue from CS-treated mice. However, CS exposure significantly decreased Agtr1a expression in our model. Similarly, Agtr2 mRNA levels were reduced in the lungs of CS-exposed mice.

A significant quantified decrease in Mac3^+^ staining in the lung tissue with losartan treatment has been demonstrated in a mouse model of COPD^25^, suggesting the amount of macrophages recruited to the lung had decreased. In contrast, losartan treatment in our model did not decrease BALF cellularity. CXCL1 increased following CS exposure with both vehicle and losartan treatment and there was no significant difference between the two groups. This is consistent with a study that found BALF levels of CXCL1 were not decreased by treatment with another RAS inhibitor, enalapril, in a model of lipopolysaccharide-induced lung injury^48^. Treatment with losartan in CS-exposed mice significantly increased CCL2 expression in the lung tissue in comparison to sham vehicle mice suggesting losartan may have been involved in the macrophage infiltration observed in our model. Interestingly, this result contrasts to the cell counts as losartan seemed to induce a slight decrease in the total number of macrophages in the BALF. The CS-induced increase in TNF-α mRNA expression in the lung tissue was unaffected by treatment with losartan; however, losartan induced a significant decrease in IL-6 expression suggesting that it may have had a small effect on lung inflammation. Finally, losartan had no effect on the weight of the lung in CS-exposed mice. As no reduction was observed in BALF cellular inflammation, chemokine, or TNF-α expression, the results of this study suggest losartan has little effect on lung inflammation in this preclinical model of COPD. It is possible that we did not see a decrease in CS-induced BALF inflammation with losartan because the receptor blockade was incomplete. However, this is unlikely given that although losartan did not reduce CS-induced lung inflammation in our study, it did increase peripheral fat accumulation (testicular fat and retroperitoneal fat), reduced CS-induced increases in lung IL-6 mRNA and rescued water intake. These observations give confidence that sufficient drug was being ingested to cause a physiological effect and that the absence of an effect on the inflammatory response was real. The dose of losartan used in our study was also based on the study by Podowski *et al*.^25^ who showed that inhibition of TGF-β signalling through angiotensin receptor blockade attenuated cigarette smoke-induced lung injury.

As losartan is an AT1 receptor antagonist, the expression of components of the RAS was measured in the lung tissue to determine the effect of CS and losartan on RAS expression. Angiotensinogen is a precursor of angiotensin-II, the main effector peptide in the system, which signals via its two receptors, AT1 and AT2. There is little known about the effect of CS and losartan on the mRNA expression of these components. Treatment with losartan had no effect on the reduced expression of the two angiotensin-II receptors observed in the CS-exposed mice. In parallel, Podowski *et al*.^25^ demonstrated no change in expression of Agtr1a following losartan treatment. The reduced expression of the angiotensin-II receptors in the lung following CS-exposure in our study may explain why losartan did not decrease CS-induced lung inflammation.

COPD is a multi-factorial disease with contributions from inflammation, oxidative stress, hormone signalling, as well as appetite^6,49^. It is well established that the nicotine component of CS suppresses appetite and causes loss of body weight in both human and animal studies^50–52^. As such, the sham mice were pair-fed in our model to control for changes in appetite and to investigate the contributions of other mechanisms in mediating body and organ weight. Regardless of pair-feeding, losartan did not affect food consumption in our model. The appetite suppression from the CS possibly overrides any differences that may have been observed in the sham groups in the absence of pair-feeding. The dose of losartan chosen for our study is within the range (3–30 mg/kg) used in a similar mouse model of COPD^25^. In addition, we trialed the 3 and 30 mg/kg doses in a pilot 4 day acute CS-exposure protocol where we found that 30 mg/kg losartan caused a small (but not statistical) decrease in total BALF cellularity in CS-exposed mice and that the CS-exposed mice treated with 30 mg/kg losartan had the largest mean percentage weight gain and water consumption compared to other CS-exposed mice (data not shown).

To explore the effect of CS and losartan treatment on overall body weight, the mice were weighed 5 days per week for the duration of the protocol. A steady increase in weight was observed in all groups; however, the CS-treated mice gained significantly less than the sham groups overall reflecting the results observed in the literature^33^. According to various studies, the weight loss observed from CS treatment is due to a greater capacity for energy expenditure and not just due to the reduced food consumption^53,54^. Losartan had no significant effect on bodyweights in either group, consistent with the literature^25,55^, although losartan-treated sham mice were 2.6% heavier than sham vehicle mice at the end of the 8 week protocol. This effect is most likely explained by the increased white adipose tissue weight (testicular and retroperitoneal fat) in the sham losartan-treated mice. This was somewhat unexpected studies in rats showed that losartan alone had no effect on baseline white adipose tissue weight, suggesting that it could be a species related phenomenon^56,57^. The CS-treated mice in our model demonstrated a significant decrease in white adipose tissue weight which is consistent with studies showing that Ang II infusion decreases retroperitoneal white adipose (RWAT) fat mass^56^. However, in that study losartan reversed the Ang II-induced decreases in RWAT fat mass which was not the case in our CS study, highlighting that differences may be a species-dependent observation. In addition, losartan significantly decreased liver weight in CS-exposed mice compared to sham vehicle, and CS-exposure significantly increased kidney weight in vehicle and losartan groups compared to sham vehicle. Body composition in COPD is somewhat complex and involves many factors including genetics, appetite, and exercise tolerance^58^. As such, patients demonstrate differential responses to CS and have been shown to both increase and decrease body weight^58,59^. In both human and animal studies, CS causes a preference towards foods high in refined sugar and fat which possibly explains the increased fat weight in some cases^60,61^. In contrast, BALB/c mice are genetically identical, and all have equal access to normal experimental chow, highlighting a possible limitation in our model.

An enzyme involved in the metabolism of losartan and other drugs, CYP2C9, has demonstrated between-patient differences in the drug pharmacokinetics and pharmacodynamics, which can result in inadequate therapeutic effect or toxicity^62,63^. Comparisons between Korean and Swedish populations demonstrated that *CYP2C9* genotype, ethnicity, and CS are major determinants of enzymatic activity^64^. Higher enzymatic activity in smokers in each population was observed, with more significant changes in the Korean population^64^. In addition, CYP2C9 has been demonstrated to be involved in two pathways that trigger the metabolic activation of polycyclic aromatic hydrocarbons found in CS^65–67^. It is thus possible that CYP2C9 enzyme is required for the metabolism of components of CS in our model and is less effective at metabolising losartan. As such, further examination of the levels of losartan metabolites in the blood system is necessary to understand how losartan was metabolised in our model.

In conclusion, 8 weeks of CS exposure in mice caused significant lung inflammation as measured by increased BALF cellularity, increased whole lung mRNA expression for pro-inflammatory cytokines, chemokines and proteases and increases in lung weight. Cigarette smoke exposure also caused significant decreases in whole lung angiotensin II receptor mRNA expression and decreases in body weight, liver and adipose tissue weight. Losartan treatment did not inhibit CS-induced BALF cellularity despite reducing whole lung IL-6 mRNA and Ang-II receptor mRNA expression. In addition, losartan was without affect on CS-induced decreases in body weight, liver and white adipose tissue mass. Thus, it appears unlikely that losartan may be a viable therapeutic to treat CS-induced lung inflammation seen in patients with COPD.

## Data Availability

All data generated or analysed during this study are included in this published article.
